# Adolescent Perspectives on the Use of Social Media to Support Type 1 Diabetes Management: Focus Group Study

**DOI:** 10.2196/12149

**Published:** 2019-05-30

**Authors:** Faisal S Malik, Neil Panlasigui, Jesse Gritton, Harsimrat Gill, Joyce P Yi-Frazier, Megan A Moreno

**Affiliations:** 1 Division of Endocrinology and Diabetes Department of Pediatrics University of Washington Seattle, WA United States; 2 Center for Child Health, Behavior, and Development Seattle Children's Research Institute Seattle, WA United States; 3 Center for Clinical and Translational Research Seattle Children's Research Institute Seattle, WA United States; 4 Division of General Pediatrics and Adolescent Medicine Department of Pediatrics University of Wisconsin-Madison Madison, WI United States

**Keywords:** type 1 diabetes, adolescents, social media, qualitative research

## Abstract

**Background:**

A majority of adolescents report the use of some form of social media, and many prefer to communicate via social networking sites. Social media may offer new opportunities in diabetes management, particularly in terms of how health care teams provide tailored support and treatment to adolescents with diabetes.

**Objective:**

The aim of this study was to explore the experiences and perspectives of adolescents with type 1 diabetes on the feasibility of social media use as a tool to collaboratively manage their diabetes with their diabetes care team.

**Methods:**

Focus groups of adolescents with type 1 diabetes were conducted in the Seattle metropolitan area in Washington State. Semistructured questions were used to elicit views around the preferred means of communication with the adolescents’ diabetes care team, how to best support diabetes self-management, and how social media could be used outside of the clinic setting by the diabetes care team to engage with adolescents with type 1 diabetes. Focus groups were audio recorded and transcribed verbatim. Qualitative content analysis was carried out, and emergent themes were subsequently mapped onto 4 domains of feasibility, which included acceptability, demand, implementation, and practicality.

**Results:**

Participants included 45 adolescents with type 1 diabetes (mean age 15.9, SD 1.7 years; 58% male; diabetes duration mean 6.2, SD 3.6 years; 76% on insulin pumps; 49% wore continuous glucose monitors; 93% reported use of social media; 84% used smartphones as the primary means for social media access). A total of 7 major topics were identified and mapped onto areas consistent with our focus on feasibility. For acceptability and demand, participants expressed how communication over social media could help facilitate (1) improved communication outside of clinic visits to optimize diabetes management, (2) independence in diabetes self-management, (3) connection to other youth with diabetes for additional diabetes support, and (4) delivery of more timely and personalized care. Addressing implementation and practicality, participants shared the need to (1) ensure patient privacy, (2) maintain professional nature of provider-patient relationship, and (3) recognize that social media is not currently used for medical care by youth with diabetes.

**Conclusions:**

Adolescents with type 1 diabetes expressed interest in the use of social media as a tool to support diabetes management and increase engagement with their diabetes care team. Specific implementation measures around privacy and professionalism should be considered when developing a social media intervention to facilitate communication between adolescents and care teams.

## Introduction

### Background

Despite significant advances in medical treatment, less than 20% of adolescents with type 1 diabetes meet recommended targets for glycemic control [1]. Poor diabetes self-care adherence and suboptimal glycemic control place adolescents with type 1 diabetes at risk for decreased quality of life because of acute (eg, diabetic ketoacidosis) and chronic (eg, blindness, kidney failure) complications [2,3]. Novel strategies to support and improve adolescent engagement and adherence to type 1 diabetes self-care are needed [2,4].

The use of social media may offer one avenue to better meet the needs of adolescents with type 1 diabetes outside the ambulatory care setting. Currently, telephone, email, regular mail, and patient portals are the primary means of communication between health care teams and patients [5,6]. However, these traditional platforms show low rates of acceptability and engagement among adolescents [7]. In contrast, social media is being used by a majority of adolescents, and many cite a preference for communication via social networking sites (SNS). Of those using social media, approximately 90% of teens share that they are on the Web several times a day or almost constantly [8].

### Potential for Social Media Use in Diabetes Management

The evolution of social media over the past decade now offers adolescents an array of potential benefits, including access to information, social support, and far-reaching communication tools that have the potential to facilitate diabetes management support outside of the ambulatory care setting. Specifically, the unique functions and features of different social media platforms provide youth a variety of perceived affordances (ie, properties of different social media apps that can be recognized by users and that can contribute to their function) to support chronic disease management [9]. For example, many adolescents with type 1 diabetes may believe that certain features of social media offer cognitive affordances, such as the ability to expand one’s learning about type 1 diabetes and how to improve self-care [10]. Other adolescents may be drawn to features of certain social media platforms that offer social affordances, including a sense of belonging to a group related to diabetes, given the interactive nature of social media [10]. These perceived affordances highlight the potential for social media to be used to provide Web-based diabetes education, peer support, and decision advice through real-time bidirectional communication in a digital environment [11]. However, before such affordances can be explored or realized, an understanding of adolescents’ experiences and perspectives regarding the feasibility of social media tools is required.

Adolescents with diabetes, in particular, appear to be ideal candidates for chronic disease management support using social media, given their extensive use of technology as part of their daily diabetes management, including devices that monitor glucose and deliver insulin, diabetes-specific apps on smartphones, and internet-enabled education and support programs [12-14]. Using more mainstream SNS that are already well integrated into adolescent daily lives may be a more suitable option for supporting diabetes management outside of the clinic setting, particularly as adolescents routinely use social media to obtain health-related information, as well as social and emotional support [15-17]. Our primary aim was to explore adolescents’ experiences and perspectives on the feasibility of using social media to collaboratively manage their type 1 diabetes with their diabetes care team.

## Methods

### Study Design

This qualitative study used focus groups to explore adolescents’ views on how social media use and communication with diabetes care teams could potentially support diabetes management [18]. Focus groups provide the opportunity to acknowledge the participants as the experts. Therefore, the results are likely to inform the development of services that could be more amenable to use by adolescents with type 1 diabetes [19]. In addition, focus groups can be particularly effective with children and adolescents, as the format reduces the pressure for a particular individual to respond, as would be the case in interviews, thereby limiting any adolescent concerns about researcher expectations [19,20]. The format also allows for spontaneous, free-flowing conversations guided by a skilled moderator. Our methods were reported using the Consolidated Criteria for Reporting Qualitative Research guidelines as a framework [21]. The Seattle Children’s Research Institute Institutional Review Board approved the study procedures and ensured that ethical principles were applied to research activities.

### Study Participants and Recruitment

A purposeful sample of adolescents with type 1 diabetes that received care at Seattle Children’s Hospital Diabetes Clinics was recruited from the Seattle metropolitan area in Washington State. English-speaking adolescents (aged 13-19 years) with a type 1 diabetes diagnosis were identified through the medical record and approached via mail and phone recruitment. No current or previous experience with social media was required for participation; our goal was to allow for a full range of viewpoints, including those of adolescents with little or no social media experience.

Participants 18 years and older gave informed written consent before study enrollment. Caregivers provided written consent, and youth participants provided written assent for participants under 18. Before focus group participation, participants completed an anonymous questionnaire to collect demographic data, information about social media use, and diabetes management. Participants were provided with food at the focus group and compensated US $50 for focus group participation.

### Facilitator Guide Development

The research team comprised of a male pediatric endocrinologist with health services and qualitative research training (FM), a female adolescent medicine physician and investigator with extensive social media and qualitative research experience (MM), a female clinical research assistant with social media and qualitative research experience (JG), a male clinical research assistant with a background in patient experience (NP), and a female research health psychologist with social media and diabetes outcomes experience (JYF).

A semistructured guide was developed by the research team to explore optimal communication with an adolescent’s diabetes care team on the basis of the key components of the Health Belief Model [22-24]. The diabetes care team was defined as all health care professionals adolescents interact with regarding diabetes management, including diabetes providers, nurses, certified diabetes educators, nutritionists, and social workers.

### Focus Groups

Five focus groups with a total of 45 participants were conducted between October and December 2016 in 3 cities in the greater Seattle area (Bellevue, Washington; Everett, Washington; Seattle, Washington). Focus groups were conducted with 8 to 10 participants per group. The Seattle focus groups took place in private conference rooms at the local children’s hospital where most youth received their diabetes care; Bellevue and Everett focus groups were held in private meeting rooms at a local library. Focus groups lasted between 110 and 120 min and were audio recorded.

Each focus group was comoderated by FM and JG. NP served as a trained note taker for all focus groups. Open-ended questions and probes were used to encourage a broad discussion about participants’ attitudes and opinions around the preferred means of communication with their diabetes care team, how to best support diabetes self-management, and how social media could be used outside of the clinic setting by the diabetes care team to engage with adolescents with type 1 diabetes. The research team conducted debriefs after each focus group, and central themes were documented in focus group summaries. Per standard qualitative methodology regarding saturation, participant recruitment ended when no new themes were identified in the debriefing sessions [25].

### Data Analysis

Audio recordings were transcribed verbatim and reviewed by the moderator (FM) for accuracy. Using open coding, members of the research team (FM, NP, and JG) reviewed observer notes and transcripts to identify emergent concepts related to social media communication between adolescents with type 1 diabetes and their diabetes care team to support diabetes management [26]. These concept codes and their definitions were then discussed with the full research team and organized into a codebook.

After assurance of a foundational codebook, transcripts were uploaded to Dedoose version 8.0 software, a Web-based app for managing, analyzing, and presenting qualitative research data [27]. A total of 2 members of the research team (FM, NP) then independently completed line-by-line open coding and applied codes to all transcripts. Questions, disagreements, and newly proposed codes were discussed and resolved through regular bimonthly analysis meetings until consensus was reached. Codes and their corresponding definitions were edited accordingly. Upon completion of all coding, the research team grouped the most significant themes and decided upon the most salient domains, which are presented in this paper. These themes were then mapped onto 4 areas of focus commonly explored by feasibility studies, including acceptability, demand, implementation, and practicality [28].

## Results

### Demographics

The study included 45 adolescents with a mean age of 15.9 (SD 1.7) years. A total of 34 out of 45 participants (76%) reported using an insulin pump, with approximately half of the participants sharing that they were using a continuous glucose monitoring system to support diabetes management. A total of 42 out of 45 participants (93%) were current users of social media, and 38 out of 45 participants (84%) used a smartphone for social media access (see Table 1).

**Table 1:** Focus group participant demographic data (N=45 adolescents).

Participant characteristics		Values
Age (years), mean (SD)		15.9 (1.7)
Gender (male), n (%)		26 (58)
Race (non-Hispanic white), n (%)		35 (78)
Parental education (college or higher), n (%)		33 (73)
Diabetes duration (years), mean (SD)		6.2 (3.6)
Use insulin pump, n (%)		34 (76)
Use continuous glucose monitor, n (%)		22 (49)
Use social media, n (%)		42 (93)
**Social media platform most often used, n (%)**		
	Snapchat	15 (33)
	Facebook	11 (24)
	Instagram	8 (18)
	Twitter	4 (9)
	YouTube	7 (16)
Use smartphone for social media, n (%)		38 (84)

### Summary of Findings

**Figure 1:**
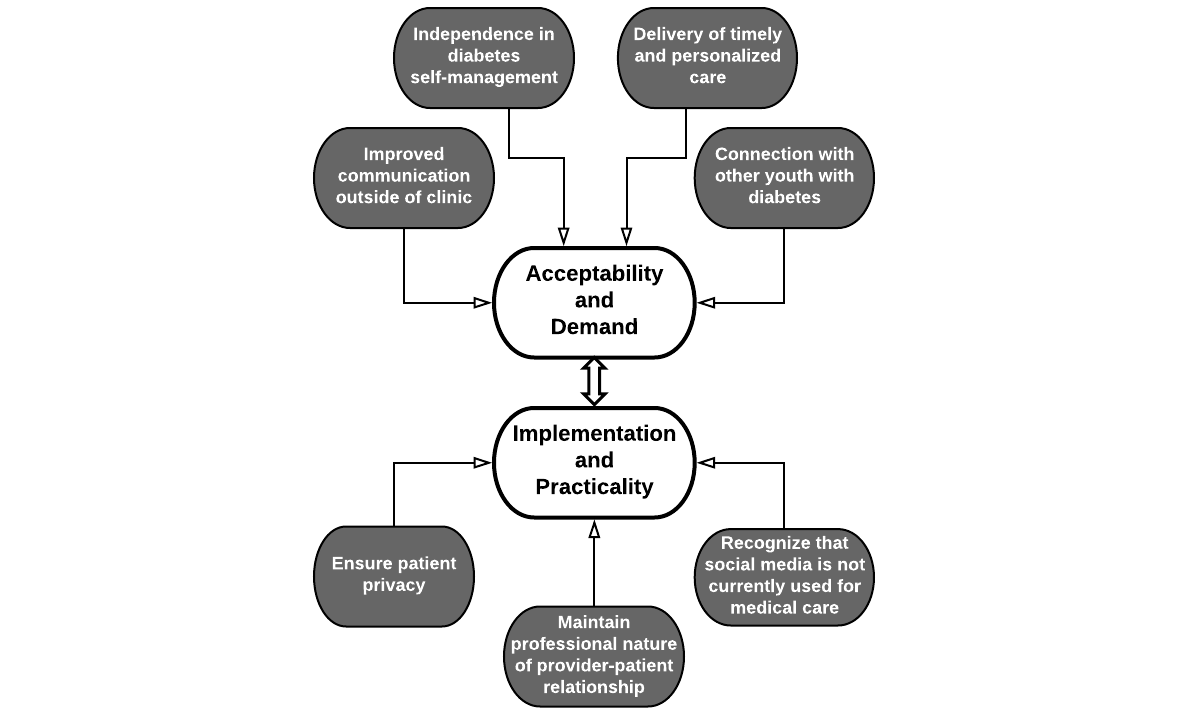
Key feasibility domains for social media use with diabetes care team.

A total of 7 major topics were identified consistent with this study focused on feasibility (Figure 1). Adolescent participants expressed that communication over social media could help facilitate (1) improved communication outside of clinic visits to optimize diabetes management, (2) independence in diabetes self-management, (3) connection to other youth with diabetes for additional diabetes support, and (4) delivery of more personalized care. In addition, participants shared important considerations regarding the implementation and practicality of developing a social media intervention for youth with diabetes, including the need to (1) ensure patient privacy, (2) maintain professional nature of provider-patient relationship, and (3) recognize that social media is not currently used for medical care by youth with diabetes. 

#### Domain 1: Acceptability and Demand

Adolescents in this study provided insights into the potential acceptability and demand for social media communication as a tool to support diabetes management and promote engagement with their diabetes care team. Participants also expressed their views regarding the suitability of and comfortability around using social media in this capacity.

##### Improved Communication Outside of Clinic Visits to Optimize Diabetes Management

For intermittent contact with the care team between clinic visits, focus group participants expressed a preference for communication over social media, compared with existing methods, such as phone or email. In comparison to email, youth stated that social media is more widely used, checked more often, and more user friendly on a smartphone. Participants highlighted the multifaceted potential of social media, noting that in addition to direct private messaging, it offers an opportunity to share diabetes-related educational content, news updates, and current research through visual means.

I don’t really check my email a ton. Social media would probably be the easiest way to get a hold of us people.

Adolescent 3, FG1

I would also say that it’s very convenient because a lot of social media [platforms] are on your phone…usually everybody has their phone on them, especially as an adult, and it’s just at the tip of your fingers. It would be a lot easier to respond…I can’t stand emailing people through my phone. I have to do it on a computer, and that’s just my personal preference, but I feel like a lot of people are like that.

Adolescent 5, FG5

Adolescents from the focus groups also shared that a designated diabetes care team contact that could be reached via social media would likely be easier to access, as they would not have to experience lengthy wait times to be triaged to the appropriate diabetes care team member as they do now when contacting the team over the phone.

I feel like I have to jump through so many hoops to get to [talk to a diabetes care team member]. The transfers and the call, or just them not being there, and just not even wanting to call because I'll know I'll have to do that.

Adolescent 1, FG2

Participants also shared that social media communication is less formal for them than having to write an email, and thus it would offer a mode of communication that is more conducive for submitting questions and brief check-ins between clinic visits. In addition, they perceived email as being overwhelmingly cluttered and felt that social media offered a more organized platform for communication.

I get a lot of emails and it’s really hard to distinguish what is spam and what is not [since] sometimes it doesn’t go into the spam box. I’ll click on something and, oh my god, it will open up something completely different, which is really complicated, especially if I am looking for an important email and I can't find it. I feel like a social media page would be a lot better because depending on how it would be set up, it would be organized.

Adolescent 7, FG1

##### Independence in Diabetes Self-Management

Youth shared that the opportunity to use social media offered a means of empowering them to be more responsible for their diabetes self-care and potentially facilitating increased autonomy.

My parents are normally the ones who would talk with the doctors and stuff and so with social media it would be like I’m taking more control over what’s happening…it’s easier to talk with the doctors and stuff instead of having to call them, find the phone number and all that stuff. You can just send them a message.

Adolescent 4, FG1

Many felt that engaging their diabetes care team through social media offered an avenue to reach out to the medical team without the help of their caregivers and better supported continued correspondence over time.

Usually when my doctor is communicating with someone in my family about my diabetes, it’s with my mother…[But] my mom doesn’t know everything that is going on during the school day or hanging out with friends with my diabetes, so it would be nice…[if] I could talk to [my provider] about what’s going on in my personal life revolving around diabetes.

Adolescent 5, FG1

In addition, participants shared that having a more direct line of communication with the diabetes care team would be beneficial, particularly for adolescents preparing for a transition to college or into the workforce. Several students graduating from high school highlighted that social media, in their opinion, could allow for a smoother transition by increasing their access to convenient remote support, especially as they attempt to navigate their diabetes self-care in a new environment without caregiver support.

##### Delivery of Timely and Personalized Diabetes Care

Participants highlighted that they were interested in engaging with their diabetes care team on social media, as they felt it would provide an opportunity to follow-up on self-management goals between visits. Although most adolescents shared that they do not communicate between clinic visits, many participants felt that through social media, they would potentially be able to meaningfully communicate about diabetes management goals by touching base at regular short intervals between clinic visits.

I know we have the goals we set at our check ups, or at our appointments, but… figuring out a short term goal for yourself, and then being like okay, we’re gonna check back two weeks from now [over social media].

Adolescent 4, FG2

In addition, adolescents expressed that the option of communicating over social media could also allow them to address acute issues that may arise between visits.

If it’s a question that affects your care during those three months [between clinic visits], or it does something to help what you’re doing within diabetes, then that could be helpful because you get the answer then and not have to wait.

Adolescent 2, FG2

Several youth felt that their relationship with the care team could potentially be enhanced by knowing who the care team members were outside of the clinic setting, which they felt could lead to increased trust and translate into more open communication. Some were eager to establish closer relationships with their providers outside of the clinic setting, whereas others could at least appreciate the efforts to build better rapport overall. 

I think you would get a more personal relationship with your doctor [through social media] without it being creepy…you would probably get to know each other a bit more and you would be more knowledgeable about things going on.

Adolescent 1, FG1

A few participants felt that a better understanding of their personal life could facilitate more personalized diabetes management recommendations to support self-care. As an example, youth mentioned that a diabetes care team member who could see the kinds of food they ate on their social media account might be able to provide better recommendations regarding insulin dose adjustments and nutrition recommendations.

[Through social media] they would know more about me, and what I like and what kind of foods I like, so that could also factor into insulin pump settings.

Adolescent 8, FG4

##### Connection to Other Youth With Diabetes to Help Optimize Diabetes Management

Most participants shared that they do not currently have a circle of friends with whom they can relate to regarding their chronic disease, and although some may know of a peer or family member who is also living with diabetes, relationships built around diabetes are not common. A majority of adolescents agreed that emotional support from peers would be valuable. Thus, many focus group participants expressed an interest in the idea of being able to engage over social media with not only their diabetes care team but also other youth with diabetes.

You can form really strong bonds over the internet and I think if you have something as big as diabetes in common then like you could probably bond really fast. I mean I trust you guys and I've only met you today.

Adolescent 4, FG1

Some participants viewed social media as an avenue for connecting with different resources and gaining new perspectives on diabetes care. These participants highlighted the potential of exchanging ideas with their peers on the Web and gaining insight into their unique care routines. Participants felt that unlike email, which limits their ability to conveniently gain input from other youth, social media would facilitate easier engagement with a larger network of peers. Several participants mentioned that simply having a credible and trustworthy social media page related to diabetes management that adolescents could access would provide the means to receive useful diabetes-related information with the freedom to engage as needed.

There should be a part on the [social media] page where...so you are seeing different perspectives, like from someone who lives on the east coast and west coast or someone who lives where there is a lot of resources and not a lot of resources. To see what is working and what’s not working because for me that would kind of open my eyes to things that are out there, what works and what doesn't work and for me I like trying new things so it would be pretty cool to see…their daily routine.

Adolescent 2, FG1

If you were talking to someone that had a certain way they handled their diabetes, that you liked...you could exchange tips and tricks for treating diabetes.

Adolescent 3, FG5

Furthermore, adolescent participants felt that the ability to use social media to support diabetes management presented a unique opportunity to build a peer community without parental involvement, and it could help them in embracing how they are different from other youth who do not have diabetes.

I feel like having a community is a huge step towards really accepting it because I feel I haven’t really accepted it yet, even though it’s been like a really long time. It’s really hard to get used to, and there’s always ups and downs and different variables it affects. It just changes all the time, and there’s no norm, so it’s really hard to get the feel of that. I feel, like, having other people who can be a different norm with you would be a big step in helping you improve your attitude towards diabetes, and make it fun.

Adolescent 5, FG5

#### Domain 2: Implementation and Practicality

Participants also shared their views on elements related to successful implementation for using social media to communicate with their diabetes care team. Specifically, adolescents highlighted items that should be considered during intervention development to support increased comfort with social media use for diabetes management.

##### Ensure Patient Privacy

When discussing measures that would need to be taken to promote buy-in from youth with diabetes to use social media to support diabetes management, youth cited the need to safeguard private social media conversations. For example, youth highlighted they did not want their direct messages (DM) with the diabetes care team from becoming public.

In a DM [conversation] it’s only going to me ... [but] I feel like if it was me and [care team member] in a DM, I could add anyone at any time, or [the care team member] could add anyone at any time, and then it's no longer private.

Adolescent 3, FG2

A majority of participants were not worried that social media may not be secure enough to guarantee the confidentiality of sensitive information. Participants who did express concern were primarily worried that their condition would accidentally be broadcast on social media, especially to people they had not intended to share diabetes-related information publicly. Youth who did not want to participate in social media for this reason preferred the idea of texting with the diabetes care team rather than using existing methods, such as email or phone.

##### Maintain Professional Nature of Provider-Patient Relationship

Most youth specifically valued the professional nature of their relationship with their provider, which some felt could be compromised through the use of social media. Several did not want diabetes care team members commenting publicly on pictures, hemoglobin A_1c_ values, or aspects of their personal lives that may be affecting their diabetes management.

I think it would be good in some ways, but it might be awkward at the same time, like, if your doctor’s following you on Instagram and they can see everything you’re posting and all that stuff.

Adolescent 4, FG3

##### Recognize That Social Media is Not Currently Used for Medical Care by Youth With Diabetes

Participants reported that most use social media to share information about their personal lives or about things they find interesting that are usually not related to their diabetes. Although a majority of participants were avid social media users, they shared that the lack of experience using social media to supplement their diabetes management might initially be a barrier to engagement. In addition, they shared they would have to become comfortable with using social media for purposes other than social networking.

I think it would be a little weird [engaging over social media] at first. I don’t really know, I’m not very creative in thinking of how that would work but, I mean, I would do what they thought of.

Adolescent 6, FG1

## Discussion

### Principal Findings

This qualitative study found that adolescents with type 1 diabetes are interested in using social media as a tool to support diabetes management and increase engagement with their diabetes care team. However, participants also expressed some concern about certain features that are part and parcel of social media platforms. In addition to highlighting social media’s strengths as a means of communication for youth, adolescents also shared that having the option to communicate over social media has the potential to promote autonomy and facilitate timely and individualized care. Recognizing that adolescents with type 1 diabetes are at high risk for poor health outcomes [29], social media may offer a means to enhance collaboration with the care team to improve diabetes management outside of the clinic setting.

Collaborative communication between providers and patients is viewed as a key element in achieving favorable health outcomes in disease management [30]. However, challenges around adequate communication and the ability to access timely health-related information continue to be a barrier to optimal type 1 diabetes care for youth [31]. The use of technology has been recommended as an avenue for improving communication with adolescents [30]. Youth in this study provided insights into their specific communication preferences on the basis of the perceived affordances of social media, and they expressed a clear interest in using some features of social media platforms to facilitate increased contact outside of clinic visits. Adolescents with type 1 diabetes viewed social media as an avenue for more direct and timely communication with their diabetes care team than existing means. These results are consistent with trends that highlight increased use of social media communication in adolescents compared with email and voice calls [32].

In an era of increasing electronic health implementation, health care organizations are making use of patient portals to enhance patient-provider communication and support self-management [33]. Specifically, these electronic systems are being designed to allow patients to view their electronic health record and offer options to communicate with providers through secure messaging, refill medications, and schedule appointments [34,35]. However, patients report many usability barriers, such as unfamiliarity with portal features and unknown or lost log-in information [36,37]. Several studies also reveal racial and ethnic disparities in portal enrollment [33,38,39]. In this study, participants reported a high level of familiarity and comfortability with the current social media platforms and perceived many of them to be well designed for smartphone use. Given this, social media may be a more ideal method for adolescents to communicate with their diabetes care team, as it bypasses new design- and navigation-related burdens and is already well accepted and highly used among adolescents [40].

The shared responsibility model is encouraged in diabetes management, and it highlights that maturing adolescents should work toward an increasing level of responsibility in diabetes management while maintaining appropriate support from their caregiver [41]. However, there are few distinct pathways to facilitate the increase in independence for adolescents. This study’s results indicate that the availability of social media communication could be a novel means of supporting the adolescent in assuming responsibility for between-visit communication. Without the help of their caregivers, a majority of adolescents stated they would be willing to initiate direct communication with the diabetes care team about nonemergent issues related to their diabetes through social media, something they do not routinely do through the existing communication means available to them. Although caregiver involvement is considered an integral part of supporting adolescent diabetes management, the development of adolescent autonomy has been identified as an important component for successful diabetes management in adulthood, as well as overall psychosocial development in adolescents with type 1 diabetes [42,43]. Providing adolescents with an opportunity to be more independent through social media communication may also improve engagement in self-care, as higher self-reported autonomy in diabetes management has been shown to be correlated with better adherence to treatment in adolescents with type 1 diabetes [42].

Adolescents in this study felt that communicating with their diabetes care team via social media could also enhance personalized care by allowing diabetes care team members to gain a better sense of their lifestyle, engage in more short-term goal setting, and build better rapport between them. The suggestions offered by the participants reflect a desire for patient-centered communication and support, encompassing aspects such as thorough information exchange, collaborative goal setting, and shared decision making [44,45]. These strategies have been shown to be associated with better adherence to treatment and perceptions of empowerment in youth with type 1 diabetes, as they can facilitate a better understanding of the unique needs and preferences of adolescents and encourage active patient engagement [44,46].

The role of social media has expanded to health education and management in youth, with many seeking information on disease management, as well as social and emotional support on the Web [15,17]. This study’s results confirm the desire of adolescents with type 1 diabetes to obtain information related to health management through social media, not just from their diabetes care team but also from other youth with type 1 diabetes. However, as recognized by health care professionals themselves, the overall reliability and accuracy of health information accessible to patients on social media can be poor [47,48]. Involving health care professionals in a moderating role in Web-based communication among peers can assist in countering misinformation through distribution of evidence-based medical expertise and facilitate social support for patients [49]. In addition, moderators can encourage patient engagement, maintain focus in discussions, and redirect patients to additional, reliable resources [50].

A barrier to using social media for health communication is the risk for violation of patient privacy. Breaching of patient privacy can carry negative legal implications for the diabetes care team members under violations of Health Insurance Portability and Accountability Act and other professional guidelines [48,51]. In addition, some people are opposed to being portrayed as patients on social media, and they fear the publicizing of their personal health information [52]. Women, in particular, are known to highly value privacy and use restrictive privacy settings at higher rates than men [53]. Participants in this study echoed these attitudes, as they did not desire to share information about diabetes with their larger social media networks. However, participants did acknowledge the use of more private social media features, such as DM, as acceptable means of communication with their care team. Future social media health communication interventions should prioritize patient privacy and leverage social media privacy settings and features to ensure a secure environment.

Most participants in this study expressed a preference for maintaining a professional relationship with their provider. Preservation of professional boundaries on social media has also been recognized as an important issue by the medical community, as health professionals are increasingly using social media to provide evidence-based information to broad communities, network with colleagues, and engage in advocacy or public health initiatives [54,55]. In the efforts to address these concerns, professional medical organizations have published policy statements that include guidelines regarding ethics of professionalism on the Web [56].

### Limitations

This study has a few limitations. First, this qualitative study is limited by use of a sample recruited from a major metropolitan area. It is possible that the views of adolescents living in rural settings may differ, even though the option for increased remote care support may be desirable to adolescents with type 1 diabetes, as these patients often face decreased access to clinic facilities and increased transportation challenges. Second, by excluding adolescents who did not speak English, we may have missed themes of particular importance to non-English-speaking adolescents. Finally, given that the majority of the participants were already avid users of social media, this study’s results may have shown bias toward an acceptance of social media for health communication purposes. Future studies should aim to explore perceptions regarding social media communication in adolescents of varying racial, ethnic, and socioeconomic backgrounds.

### Conclusions

This study’s results suggest that the use of certain social media platform features to support diabetes management outside of the ambulatory setting is acceptable to adolescents with type 1 diabetes. Given social media’s potential to enhance communication with adolescents, the use of social media as a health collaboration tool among adolescents with type 1 diabetes should be actively considered by diabetes care teams. Although social media offers the potential to improve patient care in a multifaceted manner, elements related to successful implementation should be carefully reviewed, specifically the efforts to preserve privacy and professionalism. In collaboration with technology experts, an understanding of the advantages and disadvantages of the various social media platform features, as well as adolescents’ acceptability and enthusiasm for various affordances different platforms of social media offer, should be acquired. Finally, although it is known that a considerable amount of health care professionals already use social media in some fashion related to their field, their perspectives and concerns should be taken into consideration when further exploring the feasibility of social media as a platform for health collaboration with patients. 

## Abbreviations

DM: direct messaging
SNS: social networking sites
